# Early childhood family background predicts meal frequency behaviour in children: Five-year follow-up study

**DOI:** 10.1177/14034948211058544

**Published:** 2021-12-14

**Authors:** Suvi Parikka, Tuija Martelin, Sakari Karvonen, Esko Levälahti, Laura Kestilä, Tiina Laatikainen

**Affiliations:** 1 Finnish Institute for Health and Welfare, Department of Welfare, Finland; 2 University of Eastern Finland, Institute of Public Health and Clinical Nutrition, Finland; 3 Joint Municipal Authority for North Karelia Social and Health Services, Finland

**Keywords:** Social determinants, childhood health behaviour, survey data, prospective setting

## Abstract

**Aims:**

Childhood nutrition patterns have an important role in later health. We studied the role of family type, other family background factors and their changes over a five-year follow-up with respect to meal frequency among children.

**Methods:**

Longitudinal data were collected in 2007–2009 and 2013–2014. A nationally representative sample of Finnish children (*n* = 1822) aged 0.5–5 years at baseline and 5–10 years at follow-up and their families were used. The participation rate was 83% at baseline and 54% at follow-up. Meal frequency was defined as four to six meals per day. The associations of meal frequency with family background factors over a five-year follow-up period were examined by bivariate and multivariate regression analyses.

**Results:**

Eighty-nine per cent of the 5–10-year-old boys and girls had the recommended meal frequency at follow-up. Living in a single-parent family at baseline increased the risk of not eating the recommended number of meals compared with those living in intact families. After adjustments, a mother’s low level of education (OR 0.51, CI 0.29–0.93) and a decrease in income sufficiency (OR 0.54, CI 0.35–0.84) during the follow-up period were unfavourably associated with the recommended meal frequency. The difference between children in stable single-parent, reconstituted or joint physical custody families and those living in stable intact families remained significant when controlling for other variables.

**Conclusions:**

**Single-parent families with a low socioeconomic position represent important target groups for interventions designed to promote regular meal frequency**.

## Introduction

Low socioeconomic position (SEP) and living in a single-parent family have been suggested to be associated with irregular meal consumption in childhood [1–4]. Children’s eating behaviour, including regular meal frequency, is receiving growing attention with the rise in obesity and chronic nutrition-related diseases.

In Finland, according to the national recommendations for families with children, *Eating Together* [5], a regular meal schedule is the foundation for healthy eating for both children and adults. Small children need food frequently because they cannot and should not eat large quantities at any one time. Long intervals between meals can result in uncontrolled eating and unnecessary snacking and thus cause problems with overweight. The recommendation is that both children and adults should eat every 3–4 h, which translates into about four to six meals a day.

Children are dependent on their parents and their health behaviour is largely shaped by the material and non-material resources of their parents and family. Skipping breakfast and other meals has been associated with a low family SEP, irrespective of SEP indicator (e.g. parental education, parental occupation and household income) [1,2,6,7]. Over recent decades, family diversity has increased as the proportion of traditional intact families has decreased [8]. Although intact families still remain the most common family type in Finland, 19% of children aged 0–17 were living with only one parent and 10% were living in a reconstituted family in 2017 [9]. In general, the literature suggests that living in a single-parent or reconstituted family is less favourable for the development of a child’s health behaviour [10,11]. Children may receive less parental time, attention and material support in both these family types [12,13].

Few studies have analysed the associations between the family type and children’s daily meal frequency. Earlier findings suggest that family type has a direct and strong association with the number of meals a day among Finnish children aged 7–11 years and it also mediates the effect of parental education on meal patterns in childhood [4].

Some studies have suggested that the transition from one family form to another (e.g. from having married parents to becoming a single-parent family) rather than the family type as such may negatively affect children’s health through concomitant factors (e.g. parental conflict, loss of parental contact and reduced family income following separation) [14–16]. Previous studies have focused on parental marital status as the key indicator of family type and parental divorce as the measure of familial transition, largely due to data constraints. The findings are mixed, however, with some showing no association between family transitions and children’s physical health [17,18], while others show a negative association [14–16,19].

No study has yet been conducted on the interrelationship between the number of meals a day in childhood and family type transitions in a follow-up study setting. Furthermore, the relationships between socioeconomic factors, family type, changes in both of them and meal frequency in childhood have not previously been in the same study and thus more comprehensive investigation is needed.

The aim of this study was to assess the association of meal frequency with parental socioeconomic factors (education, labour market status and income) and family type at baseline and the changes in them over a five-year follow-up period of a cohort of children aged 5–10 years. In particular, we focused on the role of family type and its changes with respect to meal frequency.

## Methods

### Data collection

**Figure 1.**
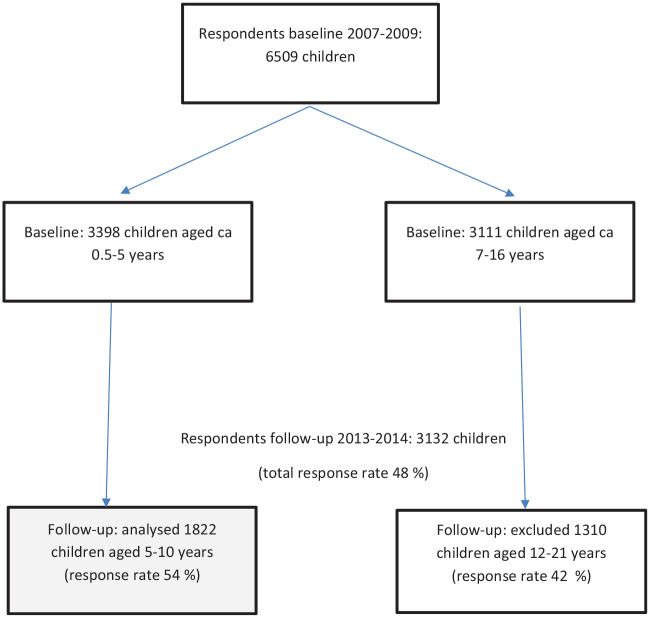
Diagram of study participants.

The data were collected in 2007–2009 and 2013–2014. The study was carried out by the Finnish Institute for Health and Welfare. In total, 6509 children participated in the baseline study. The participation rate was 83% for children aged 0.5–5 years at baseline (Figure 1).

In the follow-up-study, questionnaires were sent to all the children and families who participated in the baseline study. Both the baseline and the follow-up study consisted of a self-administered questionnaire for parents. In total, 3132 children aged 5–21 years participated in the follow-up study.

We included 1822 children (875 boys, 947 girls) aged 5–10 years at the time of the follow-up study in the analyses. The participation rate was 54%. Children aged 12 years and older at the time of the follow-up were not included in the study. This was because the response rate was low and that can lead to biased outcomes. Further, for those aged 15–21, nutrition recommendations are slightly different and not all of these young people live with their parents.

Both the baseline and the follow-up study were approved by the Coordinating Ethics Committee of Helsinki and Uusimaa Hospital District. Participation was voluntary and parents gave written informed consent before enrolment in the baseline study in 2007.

### Key variables

Meal frequency was assessed using the question ‘how often during the last week (5 days, excluding weekends) has your child eaten the following meals: breakfast, a mid-morning snack, lunch, a mid-afternoon snack, dinner, an evening snack, evening meal, other snacks?’. The responses were categorised as a dichotomous variable: the recommended number of meals (four to six meals a day) and other (less than four meals a day or more than six meals a day) according the national dietary recommendations for families with children [5].

Information on self-reported family SEP (parental education, labour market position, perceived income sufficiency) and family type was obtained twice: in the baseline study in 2007–2009 and after a five-year follow-up period. Parental education was categorised according to the highest achieved educational level: secondary education, holding a lower academic degree (bachelor’s degree) and holding an upper academic degree (master’s degree). For parental education, no change variable was calculated because education is a generally stable factor.

The parental labour market status was re-categorised into full-time employment, unemployed and other (part-time employment, being a student, stay-at-home mothers/fathers, being on military service, retirement, other). Unemployment in the family during the follow-up period was coded as: no unemployment in the family, unemployment at the time of the baseline, unemployment at the time of the follow-up and unemployment during the follow-up period. For single-parent and reconstituted families, this variable was coded solely according to cohabiting parents’ answers if the response from a non-cohabiting parent was missing. The perceived income sufficiency was based on the parents’ answers to the perceived difficulty or ease of covering family expenditure with their household income. This variable was classified into three categories: hard, quite easy and easy. Any change in the perceived income sufficiency at the time of the follow-up study compared to the baseline was coded according to the stability or change in income sufficiency into the following categories: has remained easy or quite easy, has remained hard, income sufficiency has improved, or income sufficiency has worsened.

The family type was coded as: an intact family, a reconstituted family or joint physical custody with a 50–50 schedule and a single-parent family. Joint physical custody families (*n* = 7 in the baseline study, *n* = 48 in the follow-up study) included those children living for an equal amount of time with their mother and father in two separate homes. A change in the family type noted in the follow-up study was coded into four categories: a stable intact family, a stable reconstituted family, a single-parent or joint physical custody family, a new reconstituted or joint physical custody family and a new single-parent family. At the end of the follow-up period, there were 144 single-parent families, of which 95% were single-mother families and 5% were single-father families.

### Statistical methods

Spearman’s rho correlation coefficients were calculated for the family background factors (parental education, labour market position, perceived income sufficiency, family type) at baseline and for the family background change variables for the follow-up period. In addition, multicollinearity between explanatory variables in fully adjusted multivariate models was assessed by examining the tolerance and the variance inflation factor.

There was no significant interaction between gender and the explanatory factors. Thus no gender- or age-group-specific analysis was carried out, but all models were adjusted for both gender and age group (5, 6, 8 and 10 years) of the child.

In bivariate logistic regression models performed jointly for boys and girls, the meal frequency was treated as a dichotomous outcome variable. Gender and all explanatory factors (baseline and change variables) were treated as categorical variables.

The association between potential explanatory factors and outcome variables was explored in bivariate regression analyses. Statistically significant explanatory variables (*p* < 0.05 that had any category that differed from the reference category at this significance level) were selected for further modelling. The family background factors were added to the model sequentially, starting with the family type at baseline and changes in the family type variable, followed by other parental SEP variables occurring sequentially over time (first education, then the labour market position and income together). In the final phase, all statistically significant variables were explored in a fully adjusted multivariate regression analysis. The final multivariable model was tested against the full model using a likelihood-ratio test. The results are presented as odds ratios (ORs), together with the 95% confidence interval (95% CI). The bivariate and multivariate regression analyses were performed using the SPSS (version 26) statistical program.

#### Non-response analyses

Using survey data on family background factors at the baseline, children with missing data at follow-up were compared using χ^2^ tests with children who participated in the follow-up. Data were more often missing for children with single-parents (χ^2^ = 19, *p* < 0.001), children whose mothers had a low educational level (χ^2^ = 54, *p* < 0.001), children whose fathers had a low educational level (χ^2^ = 31, *p* < 0.001), children from families experiencing income hardship (χ^2^ = 32, *p* < 0.001) and children with an unemployed father (χ^2^ = 9, *p* = 0.009). Non-responses did not differ according to the mother’s labour market position at baseline (*p* > 0.05).

## Results

According to the results, 89% of the 5–10-year-old boys and girls ate the recommended number of meals per day in 2013 (Table I). Among those children who did not have the recommended frequency, 8% had less than four meals a day and 3% had more than six meals a day. However, eating four to six meals a day was less common for older children: for instance, 92% of 5-year-old children had four to six meals a day while the figure for 10-year-old children was 84% (data not shown). Further, 92% of children in the recommended number of meals (four to six meals) category had at least three main meals (breakfast, lunch, dinner, supper) a day.

**Table I.** Distribution of parental characteristics and prevalence (%) of children’s recommended meal frequency in the follow-up study compared with parental characteristics for children aged 5–10 years.

	Distribution of parental characteristics (*N* = 1822)^a^	Children eating recommended meal frequency (four to six meals per day) (%)	*p* ^b^
Total prevalence (*n* (%))		1615 (88.6)	
Total *N*		1808	
**Family type at baseline**			
Intact family	1672 (92.9)	90.3	
Reconstituted or joint-custody family	26 (1.4)	84.6	
Single-parent family	102 (5.7)	76.2	
Total	1800		<0.001
**Family type at follow-up**			
Intact family	1484 (83.1)	90.4	
Reconstituted or joint-custody family	158 (8.8)	89.8	
Single-parent family	144 (8.1)	79.2	
Total	1786		0.001
**Change in family type during the follow-up period**			
Stable intact family	1464 (83.1)	90.4	
Stable single-parent, reconstituted or joint-custody family	70 (4.0)	72.9	
New reconstituted or joint-custody family	140 (7.9)	90.6	
New single-parent family	88 (5.0)	87.5	
Total	1762		0.025
**Maternal education at baseline**			
Upper academic degree	445 (25.2)	94.4	
Lower academic degree	790 (44.8)	90.2	
Secondary education	530 (30.0)	84.3	
Total	1765		<0.001
**Paternal education at baseline**			
Upper academic degree	356 (20.7)	92.1	
Lower academic degree	542 (31.5)	93.9	
Secondary education	823 (47.8)	85.9	
Total	1721		<0.001
**Maternal labour market status at baseline**			
Full-time employment	520 (28.6)	85.6	
Unemployed	86 (4.7)	82.1	
Other	1211 (66.6)	91.4	
Total	1817		<0.001
**Maternal labour market status at follow-up**			
Full-time employment	1140 (62.9)	89.8	
Unemployed	97 (5.4)	81.1	
Other	575 (31.7)	89.9	
Total	1812		0.707
**Paternal labour market status at baseline**			
Full-time employment	1522 (82.9)	90.1	
Unemployed	55 (6.9)	94.5	
Other	197 (10.2)	86.2	
Total	1774		0.265
**Paternal labour market status at follow-up**			
Full-time employment	1467 (82.9)	90.9	
Unemployed	122 (6.9)	83.5	
Other	180 (10.2)	84.8	
Total	1769		0.001
**Unemployment in the family during follow-up period**			
No unemployment	1464 (83.2)	90.5	
Unemployment at baseline	101 (5.7)	87.9	
Unemployment at follow-up	166 (9.4)	84.7	
Unemployment during the follow-up period	21 (1.2)	71.4	
Other/not known	7 (0.4)	71.4	0.001
Total	1759		
**Self-reported income sufficiency at baseline**			
Easy	428 (23.6)	90.9	
Quite easy	756 (41.8)	89.9	
Hard	428 (34.6)	85.9	
Total	1813		0.016
**Self-reported income sufficiency at follow-up**			
Easy	605 (33.5)	92.1	
Quite easy	740 (40.7)	89.9	
Hard	469 (25.8)	84.8	
Total	1818		<0.001
**Change in income sufficiency during follow-up period**			
Remained easy or quite easy	759 (42.0)	92.5	
Income sufficiency improved	377 (20.8)	89.1	
Income sufficiency worsened	431 (23.8)	86.0	
Remained hard	242 (13.4)	85.0	
Total	1809		0.001

a Data presented as *n* (%).

b Significance of the difference between categories of explanatory variable, Spearman’s rho two-tailed test.

All of the SEP and family type variables correlated with each other, with the exception of the mother’s and father’s labour market position at baseline. The correlation was strongest between the mother’s and father’s education at baseline (Spearman’s correlation coefficient 0.501, *p* < 0.01). However, a multicollinearity analysis of the explanatory variables yielded acceptable collinearity because the variance inflation factor varied between 1.02 and 1.37 in the fully adjusted multivariate model.

Associations between the SEP and family type and meal frequency in childhood were assessed using a univariate model and three multivariate models (Table II). In the age- and gender-standardised bivariate model, living in a single-parent family at baseline increased the risk of not eating the recommended number of meals per day in childhood compared with those living in intact families (Table II). For the family transition variable, only living in a single-parent, reconstituted or joint physical custody family throughout the follow-up period had a statistically significant and inverse association with meal frequency. All SEP indicators except the parental labour market position at the baseline had a statistically significant association with meal frequency in childhood.

**Table II.** Associations between the recommended meal frequency in childhood (at follow-up) and family type and indicators of socioeconomic position (bivariate and multivariate age- and gender-standardised models) for children aged 5–10 years.

	Meal frequency in 2013							
	Bivariate^a^		Model I: AGE+SEX+FT+CFT		Model II: AGE+SEX+CFT+ME+PE		Model III	
	OR (95% CI)	*p* ^b^	OR (95% CI)	*p* ^b^	OR (95% CI)	*p* ^b^	OR (95% CI)	*p* ^b^
**Family type at baseline**								
Intact family	1		1					
Reconstituted or joint-custody family	0.81 (0.27–2.40)	0.70	1.53 (0.31–7.62)	0.60				
Single-parent family	0.39 (0.24–0.65)	**<0.001**	0.76 (0.22–2.60)	0.66				
**Change in family type during follow-up period**								
Stable intact family	1		1		1		1	
Stable single-parent, reconstituted or joint-custody family	0.35 (0.20–0.62)	**<0.001**	0.39 (0.10–1.49)	0.17	0.39 (0.20–0.79)	**0.008**	0.47 (0.23–0.95)	**0.04**
New reconstituted or joint-custody family	1.04 (0.57–1.89)	0.91	1.10 (0.55–2.19)	0.79	1.13 (0.58–2.18)	0.73	1.38 (0.69–2.77)	0.36
New single-parent family	0.76 (0.39–1.47)	0.42	0.75 (0.39–1.45)	0.40	1.14 (0.51–2.57)	0.75	1.23 (0.54–2.80)	0.62
**Maternal education at baseline**								
Upper academic degree	1				1		1	
Lower academic degree	0.58 (0.36–0.93)	**0.02**			0.64 (0.38–1.10)	0.11	0.67 (0.39–1.16)	0.15
Secondary education	0.34 (0.21–0.54)	**<0.001**			0.48 (0.27–0.86)	**0.01**	0.51 (0.29–0.93)	**0.03**
**Paternal education at baseline**								
Upper academic degree	1				1		1	
Lower academic degree	1.33 (0.79–2.26)	0.28			1.72 (0.95–3.10)	0.07	1.82 (1.00–3.32)	**0.05**
Secondary education	0.54 (0.35–0.83)	**<0.005**			0.75 (0.44–1.28)	0.29	0.83 (0.48–1.43)	0.49
**Maternal labour market status at the time of the baseline**								
Full-time employed	1							
Unemployed	0.75 (0.41–1.38)	0.35						
Other	1.34 (0.92–1.94)	0.12						
**Paternal labour market status at the time of the baseline**								
Full-time employed	1							
Unemployed	1.81 (0.58–6.15)	0.29						
Other	0.66 (0.42–1.03)	0.06						
**Unemployment in the family during follow-up period**								
No unemployment	1				1		1	
Unemployment at baseline	0.87 (0.46–1.63)	0.66			0.99 (0.60–1.64)	0.97	1.13 (0.56–2.26)	0.74
Unemployment at follow-up	0.60 (0.38–0.95)	**0.03**			0.72 (0.47–1.08)	0.11	0.71 (0.42–1.20)	0.20
Unemployment during follow-up period	0.29 (0.11–0.76)	**0.01**			0.46 (0.19–1.08)	0.07	0.50 (0.15–1.64)	0.25
Other	0.24 (0.05–1.27)	0.09			0.26 (0.05–1.23)	0.09	0.16 (0.02–1.73)	0.13
**Self-reported income sufficiency at baseline**								
Easy	1						1	
Quite easy	0.85 (0.59–1.23)	0.39					0.90 (0.58–1.41)	0.66
Hard	0.58 (0.40–0.86)	**0.006**					0.60 (0.26–1.43)	0.25
**Change in income sufficiency during follow-up period**								
Remained easy or quite easy	1						1	
Income sufficiency improved	0.64 (0.42–0.98)	**0.04**					0.81 (0.42–1.54)	0.52
Income sufficiency worsened	0.51 (0.34–0.75)	**0.001**					**0.54 (0.35–0.84)**	**0.006**
Remained hard	0.46 (0.29–0.72)	**0.001**					0.99 (0.40–2.47)	0.99

a Binary logistic regression analyses with meal frequency (eating four to six meals per day versus others) as dichotomous outcome variable.

b Significance of the difference from the reference group; *p* < 0.05.

CFL: unemployment in the family during follow-up period; CFT: change in family type during follow-up period; CI: confidence interval; CIS: change in income sufficiency during follow-up period; FT: family type at the time of the baseline; IS: self-reported income sufficiency at baseline ME: maternal education at baseline; OR: odds ratio; PE: paternal education at baseline.

Models 1–3 were designed to clarify the pathways between the family background determinants. In model 1, both the family type at the baseline and a change in family type during follow-up were included in the same model. Because the association between meal frequency disappeared for both family type variables when included together in the same model, only a change in family type was selected for models 2 and 3. The association between having a stable other than intact family (single-parent, reconstituted or joint-custody family) and a lower likelihood of the recommended meal frequency in childhood remained in model 2 when the mother’s and father’s education were adjusted (Table II, model 2). The final model (model 3) was adjusted to include income sufficiency in the baseline along with all of the explanatory variables in model 2 and both income-related change variables (unemployment and income sufficiency). In model 3, a stable other than intact family type, a low level of maternal education and a decrease in perceived income sufficiency during the five-year follow-up period had an inverse association with the recommended meal frequency (four to six meals a day) for children aged 5–10 years. Regarding unemployment in the family during the follow-up period, the association with meal frequency disappeared after all adjustments. Instead, having a father with a lower academic degree was associated with a higher likelihood of having the recommended meal frequency in childhood.

Models 2 and 3 were run using family type as the baseline instead of a change in the family type variable. The results were similar concerning family SEP variables, but a single-parent family at baseline remained statistically and inversely associated with the recommended meal frequency (in model 2, OR 0.34, 95% CI 0.19–0.62, *p* < 0.001; in model 3, OR 0.40, 95% CI 0.21–0.77, *p* = 0.006, other results not shown).

In summary, after adjustments, a mother’s low level of education and a decrease in income sufficiency during the follow-up period increased the risk of not eating the recommended number of meals a day in childhood. As regards family type and its change, only the difference between children in stable single-parent, reconstituted or joint physical custody families and those living in stable intact families remained significant when controlling for other variables.

## Discussion

Using a unique population study data on a cohort of Finnish children and their families, we showed that there are socioeconomic and family type inequalities in meal frequency in childhood. After all adjustments, the recommended meal frequency (four to six meals a day) was less likely for children who lived in single-parent, reconstituted or joint physical custody families during the follow-up period compared with children in intact families. However, controlling for a change in family income attenuated the difference between intact families and those who underwent a family transition before the follow-up period. Regarding family SEP factors, a decrease in income sufficiency and a low level of maternal education had the strongest associations with meal frequency in children aged 5–10 years in the five-year follow-up period.

Our findings are contrary to those studies suggesting that family transitions negatively affect children’s health [16,19] because children in stable single-parent, reconstituted and joint-custody families were at greater risk of not receiving the recommended frequency of meals. The result that a decrease in perceived income sufficiency is an important determinant in stable single-parent families is in agreement with previous studies. For example, single-parent families have reported the cost and sole responsibility for preparing the meal as major barriers to family meals [20,21]. In stable reconstituted and joint physical custody families, children receiving less parental time and support could be a possible explanation for the identified relationship. In these families, children often live with step-parent, a step-sibling(s) or a half-sibling(s), which adds family complexity and might also lead to poorer parental cooperation [13,22]. Moreover, a low income is known to be related to stress and strain, which, in turn, may affect family functioning [23]. For example, working single mothers have reported significantly higher financial stress than non-single working mothers [24].

Low levels of maternal education were associated with higher risks of non-recommended meal frequency in childhood. Taking into account children’s age, gender, family type, other SEP variables and changes in family factors did not change the result. Because missing data for children at the follow-up was more pronounced among the disadvantaged families, it may have resulted in an underestimation of SEP inequalities [25,26]. However, our results confirm the findings in earlier studies in which a low level of parental education was associated with meal skipping in childhood [2,7]. The finding that children whose fathers had a lower academic degree were more likely to meet the recommended meal frequency was an unexpected finding for which we currently have no explanation.

The main strengths of the present study are the unique follow-up survey data, including a large age range and a wide variety of data on family-related issues. Although the study sample is not a national random sample, it covers different geographical areas and socioeconomic groups in Finland, making it reasonably representative of the Finnish child population. Furthermore, to our knowledge, no other study has been conducted on the interrelationship between SEP factors, family type, changes in both of them and meal frequency in childhood in a prospective setting.

There are also limitations to the current study. First, as more single-parents were lost at the follow-up compared with those who responded, we were unable to analyse stable single-parent families as an individual group due to the small number of cases. Second, the data do not include information on family functioning. Psychosocial factors such as parental stress or lack of social support might also have been useful to include in the study setting. Third, our 5-year follow-up period might also be too short to show the accumulation effect of family transitions during the follow-up period on children’s meal frequency. This might partly explain why no significant association between a new single-parent family and the recommended meal frequency in children was found.

## Conclusions

After adjustments, a low level of maternal education and a decrease in income sufficiency during the follow-up period increased the risk of not eating the recommended number of meals a day in childhood. As regards family type and its change, only the difference between children in stable single-parent, reconstituted or joint physical custody families and those living in stable intact families remained significant when controlling for other variables. In addition, single-parenthood at baseline was unfavourably associated with the recommended meal frequency.

The findings indicate that single-parent families with a low SEP background represent important target groups for interventions designed to promote regular meal frequency.
